# Effects of Dietary Copper Sources and Levels on Liver Copper Metabolism and the Expression of Transporters in Growing Pigs

**DOI:** 10.3390/ani15040526

**Published:** 2025-02-12

**Authors:** Rui Sun, Meng Li, Tianrui Zhang, Wenyan Yang, Lianyu Yang

**Affiliations:** 1 College of Animal Science and Technology, Jilin Agricultural University, Changchun 130118, China; sr1399919804@163.com (R.S.); 15500270655@163.com (M.L.); zhangtianrui2020@163.com (T.Z.); 2 Ministry of Education Laboratory of Animal Production and Security, Changchun 130118, China; 3 Jilin Provincial Key Laboratory of Animal Nutrition and Feed Science, Changchun 130118, China

**Keywords:** Cu-AA, CuSO_4_, growing pigs, Cu transporters

## Abstract

Research on the effects of organic and inorganic Cu sources on metabolic processes and mechanisms in pigs is lacking. This study investigated the effects of different copper (Cu) sources and levels on hepatic Cu metabolism and transporter factors in growing pigs. Sixty healthy piglets (initial body weight 14.00 ± 0.30 kg) were randomly divided into four groups with five replicates of three pigs each. Four diets (AM, AH, BM, and BH) had different Cu sources [Cu sulphate (CuSO_4_): A and Cu amino acids (Cu-AA): B] and levels [supplemented (120 mg/kg DM): M, supplemented (240 mg/kg DM): H]. The pre-feeding period was 7 days, followed by a 45-day feeding period. Slaughter and sample collection were carried out on the 46th day of the formal feeding period. Significant differences were considered at *p* < 0.05. The final weight and average daily gain (ADG) of growing pigs in the Cu-AA groups were significantly higher than those in the CuSO_4_ groups. Serum Cu increased with increasing Cu supplementation on days 20 and 40. Cu concentrations in muscle, liver, and liver subcellular organelles were higher in Cu-AA groups. In the CuSO_4_ groups, Cu concentrations were higher in kidneys and faeces. In Cu-AA groups, both the Cu concentrations in lysosomes and cytosol were higher, and the activities of cathepsin D (CTSD), β-glucosidase (BGL), and acid phosphatase (ACP) in lysosomes and cytoplasm were higher. Comparisons between groups showed that liver mRNA of copper transporter protein 1 (CTR1), ATPase copper-transporting beta (ATP7B), ceruloplasmin (CP), antioxidant protein 1 (ATOX1), and metallothionein (MT) was lower in the CuSO_4_ group than in the Cu-AA group, with the best performance at 120 mg/kg Cu. mRNAs for ATPase copper-transporting alpha (ATP7A), cytochrome c oxidase copper chaperone 17 (COX17), and copper chaperone for superoxide dismutase (CCS) showed a decreasing trend in the Cu-AA groups. Cu-AA is better for Cu deposition, enhances the utilisation of Cu, reduces Cu excretion, and promotes the expression of relevant enzymes and transporters in the liver.

## Simple Summary

Organic and inorganic copper sources have different characteristics and uses in animal husbandry. Organic Cu, like Cu amino acids, is favoured in pig diets for its high bioavailability and lower excretion. In contrast, inorganic Cu may be better in terms of cost-effectiveness and ease of production. However, their differences in specific metabolic processes and mechanisms are seldom reported. Past studies on copper levels mainly focused on high copper addition, overlooking the effects of copper sources and levels on copper metabolism, homeostasis, and body physiological functions. In this study, we analysed the reasons for the differences in liver copper metabolism and transporter indexes caused by dietary copper sources and levels in growing pigs and established a correlation; this aims to provide a basis for the rational use of copper sources and precise pig nutrition strategies and to explore sustainable, efficient, and economical feed solutions for pig farming, ensuring pigs’ healthy growth and production potential.

## 1. Introduction

Copper (Cu) is an essential trace element for animals and humans and plays critical roles as a cofactor of specific enzymes and an activator of biochemical processes and cellular metabolisms [1,2]. Cu deficiency impacts animal growth and development [3]. Excessive Cu accumulation is highly toxic and can cause diseases like Wilson’s disease and Menkes disease [4]. The risks of both deficiency and excess come from disrupting normal physiological functions depending on appropriate Cu levels in the body. Precise control of Cu absorption, distribution, and excretion is the key to maintaining homeostasis [5]. Related studies have shown that when piglets are fed a diet containing 125 mg/kg of Cu methionine (Cu-Met) or Cu sulphate (CuSO_4_), their growth performance is significantly improved in terms of average daily gain, average daily feed intake, and final body weight [6]. It has also been reported that compared with the CuSO_4_ group at the same Cu level, the addition of 150–170 mg/kg of Cu(HMTBa)_2_, a Cu methionine hydroxy analogue chelate, can promote the growth of nursery pigs [7]. These results confirm the rationality of the EU-recommended Cu addition level for piglets (5–170 mg/kg). They also indicate that there are differences in the effects of organic Cu and inorganic Cu on animal metabolism, which require in-depth research. Dietary Cu enters the liver via the digestive tract and portal vein and is absorbed and transported to subcellular organelles for storage. The Cu level in the liver reflects the dietary supply and absorption capacity. Different species have different tolerance to dietary Cu in the liver. Studies have demonstrated that rats have the highest tolerance to Cu in the liver [8,9], followed by chickens [10,11], pigs [12], rabbits [13], cattle [14,15], and sheep [16,17] which have a relatively weaker tolerance, possibly because of differences in Cu transport among animals.

Copper Transporter protein 1 (CTR1), essential for Cu uptake in eukaryotic cells, mainly governs the rate and amount of Cu ions entering cells, which is important for preventing Cu toxicity and maintaining homeostasis [18,19]. After Cu enters cells via CTR1, it binds to chaperone proteins, such as cytochrome c oxidase copper chaperone 17 (COX17), copper chaperone for superoxide dismutase (CCS), and antioxidant protein 1 (ATOX1). Research indicated that the abnormality of the COX17 gene can disrupt liver glucose metabolism, leading to blood glucose fluctuations and growth retardation [20,21]. When the CCS gene was knocked out in mice in vivo, superoxide dismutase 1 (SOD1) activity and sensitivity of female fertility to superoxide generators decreased significantly [22]. ATOX1 bound to Cu-dependent transport proteins and transported Cu to ATPase copper-transporting alpha (ATP7A) and ATPase copper-transporting beta (ATP7B) [23]. At high intracellular Cu levels, ATP7A and ATP7B act as ATP-dependent transmembrane translocators that facilitate the transmembrane transport of Cu ions, and in the liver, ATP7B is usually the predominant form of Cu ion transport [24,25]. The extracellular Cu transport protein ceruloplasmin (CP) secreted by the porcine liver can efficiently transport Cu ions to tissues and organs and participate in synthesising various enzymes and iron metabolism [26,27]. Metallothionein (MT) binds to Cu ions and transports them in storage and metabolism. When cells are damaged or apoptotic, the MT–Cu-ion complex is released extracellularly because of cytotoxicity, which affects the extracellular Cu ion concentration and distribution [28].

Over the past two decades, differences in Cu sources in livestock farming have gained attention. Organic Cu sources are better than inorganic sources regarding bioavailability and mineral excretion. For example copper citrate (CuCit) [29], copper proteinate (Cu-pro) [30], copper lysine (Cu-Lys) [31], and Cu-Met [32] can replace CuSO_4_ in the diet, effectively promote the growth of weaned piglets, and reduce Cu excretion. The absorption of Cu in the intestine mainly occurs through two mechanisms. Inorganic Cu is absorbed through passive diffusion, and active transport is mediated by specific transporters such as CTR1. In contrast, organic Cu complexes (such as Cu-AA) are absorbed more effectively due to their better stability and compatibility with the intestinal transport system. These differences are derived from the affinity of different chemical structures and Cu forms for the intestinal absorption mechanism [33,34]. Organic Cu is highly popular in pig feed, thanks to its advantages such as reducing Cu emissions and pollution through higher absorption efficiency, enabling pigs to convert feed more efficiently and thus saving resources, and its complex metabolism is beneficial for pig health. However, the metabolic process of organic Cu in pigs is relatively complex. Research on the effects of organic and inorganic Cu sources on specific metabolic processes and mechanisms in pigs, as well as the possible asynchrony of their biological efficiency mechanisms, is lacking. Thus, this study aimed to investigate the effects of dietary Cu sources and levels on Cu metabolism and transport factor indicators in the liver of growing pigs and analyse their effects on Cu uptake, distribution, and excretion in the liver.

## 2. Materials and Methods

### 2.1. Animal Care and Experimental Design

The experimental protocol was appropriately approved by the Institutional Animal Care and use Committee of Jilin Agriculture University in Changchun. For this research, a 2 × 2 factorial treatment design was adopted. The primary factors were Cu sources and dietary Cu levels. Sixty 45-day-old growing pigs (Duroc × Landrace × Yorkshire) with comparable body weight (14 ± 0.30 kg) were randomly assigned to four dietary treatment groups, with five replicates of three pigs each. Four diets (AM, AH, BM, and BH) had different Cu sources [Cu sulphate (CuSO_4_): A and Cu amino acids (Cu-AA): B] and levels [supplemented (120 mg/kg DM): M, supplemented (240 mg/kg DM): H]. The basal diet composition is presented in Table 1. It complies with the NRC (2012) standards for growing pigs. Except for the variations in Cu sources and levels, the compositions of other ingredients in each group are identical. For Cu-AA, Availa-Cu 10 (Zinpro, Eden Prairie, MN, USA), which is a Cu-based feed additive with 98% purity, was used. Unless otherwise specified, analytical grade CuSO_4_ and other reagents were purchased from Sigma-Aldrich.

### 2.2. Feeding Management and Growth Performance

After a 7-day pre-feeding stage, formal feeding lasted 45 days following commercial pig-feeding practices. (During the pre-feeding period, pre-feeding is carried out in a gradient substitution manner. Each stage lasts for two days, with substitution amounts of 30%, 60%, and 100%, respectively, for feeding.) During the test period, all pigs had ad libitum access to water and feed, and were fed three times a day, in the morning, at noon, and in the evening. The daily feed intake was recorded. At the end, the pigs’ weight, feed consumption, ADG, and ADFI were measured. Calculation formula: ADG = (average weight at the end of the pen-average weight at the beginning of the pen)/(number of trial days × number of pens); ADFI = total feed intake per pen/(number of trial days × number of pens); and F:G = ADFI/ADG.

### 2.3. Cu Concentrations in Serum, Tissues, and Blood Biochemical Parameters

Blood samples were collected from the anterior vein on days 1, 20, and 40. These samples were then centrifuged and stored at −20 °C for the measurement of serum Cu. The concentrations of total protein (TP), albumin (ALB), aspartate aminotransferase (AST), and alanine aminotransferase (ALT) in the serum were measured using the Drew Trilogy fully automated biochemical analyser. We slaughtered five pigs per group on the 46th day of the formal feeding period. Fresh liver, kidney, muscle, and faeces samples were collected and stored at −80 °C. Tissue samples (0.3 g each) were separately ground into powder, and 0.3 mL serum samples were taken. These were placed into conical flasks, respectively. Subsequently, 2 mL of perchloric acid (HCLO_4_) and 5 mL of concentrated nitric acid (HNO_3_) were added. The flasks were then placed on an electric hot-plate and heated for digestion until a small amount of white smoke emerged and the solution became clear and transparent. After cooling, 2.5 mL of 10% HNO_3_ was added. The solution was transferred to a 25 mL volumetric flask and diluted to the mark with deionized water. The Cu concentrations in the samples were determined by a flame atomic absorption spectrometer (AAnalyst-800, PerkinElmer, Inc., Shelton, CT, USA).

### 2.4. Enzyme Activity in Liver Sub-Organelles

The 0.2 g liver tissue samples were rinsed in 10 mL saline to remove blood, cut into 1 mm^3^ pieces, and homogenized into 10% homogenate with a glass homogenizer. Pre-cooled homogenization medium (pH 7.4, 0.01 mol/L Tris-HCl, 0.01 mol/L EDTA-2Na, 0.01 mol/L sucrose, 0.8% NaCl, 9-fold the tissue volume) was pipetted in. The homogenate was centrifuged twice at 2000× *g* for 10 min at low temperature to remove fat and connective tissue. Then, differential centrifugation (9000× *g*/min for 10 min, 30,000× *g*/min for 15 min, 108,000× *g*/min for 60 min) separated mitochondria, lysosomes, and cytoplasm. Activities of cathepsin D (CTSD), β-glucosidase (BGL), and acid phosphatase (ACP) in liver subcellular organelles (cytoplasm and lysosomes) were measured with an ELISA kit (Mlbio, Shanghai, China) following the manufacturer’s instructions.

### 2.5. Total RNA Extraction and Real Time PCR

Total liver RNA was extracted using the RNAiso Plus (AMS, Lugano, Switzerland) reagent. The integrity of the RNA was examined by agarose gel electrophoresis. The extracted total RNA served as a template and was reverse transcribed into cDNA according to the instructions of the reverse transcription kit (Takara, Liaoning, China). Primers were designed with the molecular biology software Primer 3.0. The primers were used: CTR1, 5′-CTTCTCATCATCACCCAACCTCT-3′ and 5′-CACCAAACCAGCAAACAATACTTC-3′; ATP7B, 5′-ATGGTGTGCTCTGTGGGATG-3′ and 5′-TCTGGCTGTCTTGTGGTTGTCT-3′; ATP7A, 5′-TCAGGTTGGCATTACTAAGGTGTTT-3′ and 5′-TACCCGTTTCCCCTCCTCTT-3′; CP, 5′-CATCTTGGCATTCTAGGTCCTGTC-3′ and 5′-CCTCCTGGCTCTTACTGACTCTC-3′; ATOX1, 5′-AGTCACTCGGGTCCTCAACAAG-3′ and 5′-CGCTGTGCTCAGAGTCAATGC-3′; MT1A, 5′-AACTGCTCCTGCCCCACAG-3′ and 5′-TGGGCACACCTGGCACAG-3′; COX17, 5′-CCCTGCCCCGCCTGAATC-3′ and 5′-AGGTGTCCACAGTGCTCTTCTC-3′; CCS, 5′-GCGAGACTGCCTGCATGTTAG-3′ and 5′-TCCAACTGTACCTCCACACTCTG-3′; reference gene: β-actin, 5′-CGGCATCCACGAAACTACCT-3′ and 5′-CGTGATCTCCTTCTGCATCCT-3′. RT-PCR was performed with cDNA as a template, using SYBR Green I stain (Takara, Liaoning, China) and the Step One Plus real-time PCR system (Thermo Fisher, MA, USA). The running conditions were as follows: initial denaturation at 95 °C for 5 min, followed by 40 cycles (95 °C for 15 s, 58 °C for 30 s, 72 °C for 30 s), and final extension at 72 °C for 5 min. The mRNA expression levels of each gene in liver tissue were calculated according to the 2^−∆∆Ct^ method.

### 2.6. Statistical Analyses

Data were analysed based on the model Yijk = µ + αi + βj + αi × βj + εijk. Here, µ represents the population mean, αi indicates the impact of Cu sources (with i = 1, 2), βj denotes the effect of Cu concentrations (where j = 1, 2), αi × βj represents the interaction between Cu sources and concentrations, and εijk stands for the residual effect. The data analysis was carried out using SPSS version 27.0 (IBM, Corp., Armonk, NY, USA). A two-way ANOVA was employed to analyse the effects of Cu sources, concentrations, and their interactions. Multiple comparisons using the Tukey method were used to examine the differences among the four treatments. Statistical significance was determined when *p* < 0.05. The results were presented as the mean and the standard error of the mean.

## 3. Results

### 3.1. Growth Performance of Growing Pigs

In Table 2, compared to the CuSO_4_ groups, the growing pigs in the Cu-AA groups had significantly higher final weights and ADG (*p* < 0.05). Among the four dietary conditions, significant differences (*p* < 0.05) were found in final weight and ADG, with no interaction effect. Specifically, the final weight of pigs in the AH group was notably lower than that in the BM and BH groups. Meanwhile, the mean ADG of the BH group was significantly greater than that of the AM and AH groups.

### 3.2. Serum, Tissue, and Faeces Cu Concentrations

As Cu addition increased (Table 3), serum Cu concentration significantly rose on days 20 and 40 (*p* < 0.05). Among the four dietary conditions, the Cu concentrations in the AH and BH groups were significantly higher than those in the other groups (*p* < 0.05). As depicted in Table 4, in growing pigs, the liver and muscle Cu concentrations in the Cu-AA groups were significantly higher than those in the CuSO_4_ groups (*p* < 0.05), while the kidney and faeces Cu concentrations were significantly lower (*p* < 0.05). The changing trend of tissue Cu concentrations was similar to that of serum Cu as the Cu addition increased. There were significant differences in tissue Cu concentrations under different dietary conditions. Under the combined action of the Cu sources and the Cu levels, the Cu concentrations in various tissues changed significantly. There was an interaction between the Cu sources and the Cu levels (*p* < 0.05). The liver Cu concentrations in the AM group were significantly lower than those in the BM group, and kidney and muscle Cu concentrations were significantly higher (*p* < 0.05). The liver and muscle Cu concentrations in the AH group were significantly lower than those in the BH group, and faeces Cu concentrations were significantly higher (*p* < 0.05). Notably, in the muscles of the AM and AH groups, the changing trend of Cu concentrations with increasing Cu addition was opposite to that in the BM and BH groups when comparing different Cu sources (*p* < 0.05).

As shown in Table 5, with the increase in the number of feeding days, on the 40th day, the concentrations of ALB, TP, AST, and ALT in the blood of growing pigs in the Cu-AA groups were significantly higher than those in the CuSO_4_ groups (*p* < 0.05). In the 240 mg/kg Cu groups, the concentrations of TP, AST, and ALT were significantly higher than those in the 120 mg/kg groups, while the ALB concentration was significantly lower than that in the 120 mg/kg groups (*p* < 0.05). There was significant interaction between the Cu sources and levels. When comparing the four groups of diets, the ALB concentration in the AH and BH groups was significantly higher than that in the AM and BM groups on the 20th day, but the opposite trend was observed on the 40th day (*p* < 0.05). The trends of TP and ALT were opposite to those of ALB. The AST concentration in the AH and BH groups was significantly higher than that in the AM and BM groups on both the 20d and 40d (*p* < 0.05).

### 3.3. Liver Subcellular Cu Concentrations and the Activities of Related Enzymes

The Cu concentrations in liver subcellular organelles of growing pigs were analysed (Table 6). The mitochondrial, lysosomal, and cytoplasmic Cu concentrations in the Cu-AA groups were higher than those in the CuSO_4_ groups (*p* < 0.05). With the increase in Cu addition, the change trend of Cu concentrations in subcellular organelles was similar to that in the liver. Under the combined action of the Cu sources and the Cu levels, there was an interaction between the two factors in cytoplasm Cu concentrations (*p* < 0.05). When the four dietary conditions were compared, the Cu concentrations of liver subcellular organelles differed significantly among the groups, with the BH group having the highest Cu concentrations (*p* < 0.05).

In Table 7, for growing pigs, ACP activities in the cytosol of liver subcellular organelles of the Cu-AA groups were higher than those in the CuSO_4_ groups (*p* < 0.05). The activities of CTSD, BGL, and ACP in the lysosomes and cytosol of the 240 mg/kg Cu group were higher than those in the 120 mg/kg Cu group (*p* < 0.05). Under the four feeding conditions, the activities of the three enzymes in lysosomes and cytosol showed significant differences. The activities of CTSD and BGL in the AH and BH groups were significantly higher than those in the AM and BM groups (*p* < 0.05). There were significant differences in the ACP activities in lysosomes among all groups, with the BH group having the highest ACP activity (*p* < 0.05).

### 3.4. The mRNA Expression Levels of Transporters in the Liver

As shown in Table 8, with increasing Cu supplementation, the mRNA expression levels of CTR1, ATP7B, CP, and MT in the liver decreased significantly (*p* < 0.05), while the mRNA expression levels of ATP7A, ATOX1, CCS, and COX17 were not significant (*p* > 0.05). When comparing the four dietary conditions, the mRNA expression levels of CTR1, ATP7B, CP, and MT were significantly higher in the AM and BM groups than in the AH and BH groups (*p* < 0.05).

## 4. Discussion

Cu is essential for animal health and growth and is used as a growth promoter in pig diets. Previous studies have shown that dietary Cu doses affect animal growth and organ Cu accumulation [35,36,37]. With the continuous deepening of research, it has been gradually discovered that there are differences in the metabolic pathways and bioavailability between organic and inorganic Cu [38,39,40]. Research has found that compared with CuSO_4_, the addition of 150 mg/kg of Cu methionine hydroxy analogue chelate (Cu (HMTBa)_2_) significantly increased the ADG of piglets and the Cu concentrations in the liver [41]. Meanwhile, for pigs weighing 5–25 kg, the linear slope of the feed-to-gain ratio when using Cu (HMTBa)_2_ was 2.1 times higher than that of CuSO_4_ [42]. Coffey et al. showed that pigs fed with Cu lysine complex had better ADG and ADFI than those fed with CuSO_4_. When the Cu addition level reached 200 mg/kg, the liver Cu concentrations of pigs fed with CuSO_4_ was significantly higher [43]. Therefore, the effects of Cu sources and addition levels on Cu deposition in pigs, animal growth, and health status are multifaceted and complex. This experiment further investigated their effects on Cu metabolism and transport in the livers of pigs and analysed the differences. The experimental results show that, compared with the CuSO_4_ groups, adding an appropriate amount of Cu-AA to the diet can promote the final weight and ADG of growing pigs, which is similar to the results of previous studies [44,45,46]. This indicates that Cu-AA can enable pigs to grow faster and heavier within the same feeding period. For the pig farming industry, this is conducive to improving the breeding efficiency. At the same time, the stable chelated structure of Cu-AA can affect the release and absorption of Cu in the bodies of pigs [29,41].

The Cu concentrations in the liver, kidney, other tissues/organs, and serum are key Cu metabolism indicators that ensure an organism’s health and normal physiological functions. In this study, it was found that the liver, kidney, muscle, and serum Cu concentrations of growing pigs increased significantly with an increase in dietary Cu levels. Compared to the CuSO_4_ groups, the Cu-AA groups had a rise in liver and muscle Cu and a decline in kidney and faeces Cu. This indicates that Cu-AA can promote the absorption and utilization of Cu in pigs, enabling Cu to better participate in physiological processes such as protein synthesis, which is conducive to the muscle growth of pigs and the maintenance of their overall physiological functions. Research has shown that the organic ligands in Cu-AA can interact more effectively with specific receptors or transporters on the surface of liver cells (such as Ctr1). Meanwhile, there are differences in metabolic utilization and storage, and it can more effectively participate in the synthesis of Cu-containing enzymes [47,48]. As for the decrease in Cu content in the kidneys and faeces, it is necessary to consider the filtration and reabsorption functions of the kidneys [49]. Research indicates that, compared with CuSO_4_, organic Cu has good solubility in the weakly alkaline environment of the intestine. Inorganic Cu ions mainly rely on some non-specific ion channels or transporters. The transport efficiency of these pathways is relatively low, and they are prone to competitive inhibition by other ions [50,51]. Studies have shown that excessive Cu intake can lead to a large accumulation of Cu in organs such as the liver, potentially triggering Cu-related toxicity. To verify the impact of the Cu addition levels in this experiment on the health of pigs, we measured the levels of TP, ALT, ALB, and AST in the serum. These indicators are of great significance for evaluating liver function and health. They can reflect the protein metabolism and nutritional status of the organism, and their levels change significantly with the state of the liver. Therefore, they are often used as sensitive markers of liver injury. The experimental results showed that the concentrations of TP, ALB, and AST in the serum of the 240 mg/kg Cu groups were higher than those in the 120 mg/kg groups, indicating that high-dose Cu has a stress effect on hepatocytes. Some research has demonstrated that even 1% liver injury can cause a sharp increase in AST and ALT in the serum. However, this does not necessarily imply severe liver lesions. It may also be an adaptive response of the liver to changes in Cu intake [52]. Since the liver’s tolerance to Cu is influenced by multiple factors such as pig breed, growth stage, and the content of other nutrients in the feed, and considering that this study only detected some serum indicators, the assessment of liver Cu toxicity may be incomplete. Meanwhile, taking into account that although Cu-AA is prone to deposition in the liver, its overall metabolic impact is complex, and currently, the specific molecular mechanisms of the impact of Cu sources on metabolism remain unclear, with different results under different experimental models and conditions. Therefore, it is necessary to conduct in-depth research on the detailed processes of different Cu sources in the in vivo metabolic pathways and their potential effects on other physiological functions of the organism.

The study found that elevated hepatic Cu levels caused Cu ions to enter hepatic cells and subcellular organelles along the gradient, increasing the Cu concentrations [51,53]. Normally, Cu in liver subcellular organelles is distributed such that microsomes and nuclei account for approximately 10% of the total cellular Cu, mitochondria and lysosomes for 10–20%, and the cytoplasm for approximately 60–70% [54,55]. Similar results were obtained based on previous studies by Goldfischer et al. [56]. This experiment revealed that as Cu concentrations rose, the Cu concentrations in the mitochondria, lysosomes, and cytoplasm of the liver subcellular organelles of growing pigs increased, and variations were observed among Cu sources. However, compared with the lysosomes and the cytoplasm, no similar interaction was observed in the mitochondria. This may be caused by the unique physiological environment and functional needs of mitochondria. Their redox and energy metabolisms have a high specificity for Cu ion utilisation, preventing Cu from reacting in other forms or pathways [57]. In this study, we examined the activities of key acid phosphatases, such as ACP, CTSD, and BGL, in lysosomes and the cytoplasm. ACP is crucial for the hydrolysis of surface phosphate esters of exogenous xenobiotics, the metabolism of phosphatase groups and phosphomonoesterases, as well as the digestion of intracellular biomolecules in acidic environments [58]. CTSD participates in intracellular protein metabolism [59], while BGL plays a vital role in the catabolism of carbohydrates by breaking down complex glycoside compounds into monosaccharides and other components [60]. These enzymes perform specific physiological functions within the cell, and changes in their activities can reflect the impact of Cu on the cellular metabolic processes. The experimental results showed that as the Cu level increased, the activities of these enzymes in the cytoplasm and lysosomes increased significantly. The activities in the Cu-AA groups were higher than those in the CuSO_4_ groups, and there was no interaction effect between the two factors. This is consistent with the findings of this study, which indicated that the Cu concentrations in the cytoplasm and lysosomes of the Cu-AA groups were significantly higher than those of the CuSO_4_ groups. Detecting the activities of these enzymes is helpful for exploring the changes in the lysosomal environment, pH value, and cell permeability, as well as the interaction between amino acid groups and Cu ions. This provides clues for the mechanism of copper’s action within the cell [61,62]. Moreover, the changes in enzyme activities can complement the integrity of research on Cu metabolism and transport, facilitating in-depth studies on the effects of Cu sources and addition levels on Cu metabolism and transport in the livers of pigs, and contributing to a comprehensive evaluation of the role of Cu in liver health.

In Cu metabolism, transporters are crucial for intracellular and intercellular Cu transport, because they recognise and bind Cu ions for transmembrane transport [63]. Several recent studies have focused on understanding the functions of Cu transport and chaperone proteins. CTR1 is essential for cell metabolism and transports Cu ions into cells [64]. Studies have revealed that an appropriate amount of Cu promotes CTR1 expression, whereas excess Cu inhibits its transport activity [65]. Similar trends were observed in this study. The addition of 120 mg/kg Cu to the feed enhanced the expression of CTR1 mRNA in the liver, whereas 240 mg/kg Cu inhibited it, which was related to the cell self-protection mechanism. When there is intracellular Cu overload, the organism may use negative feedback to inhibit Cu-related transporter expression, decrease Cu ion influx and abnormal distribution, and prevent cell damage from the overload [62]. The elevation of intracellular Cu levels leads to the endocytosis and degradation of CTR1 on the cell membrane, suggesting that cells regulate Cu uptake to prevent cell damage caused by excessive Cu. Cu ions transported into liver cells via CTR1 mainly have the following destinations: some Cu ions bind to intracellular Cu chaperone proteins. For example, COX17, CCS, and ATOX1 can transport Cu ions to specific sites, such as the mitochondria and Golgi network. COX17 binds to CCO with the aid of mitochondrial membrane proteins and transports Cu ions into the mitochondria, providing Cu materials for the synthesis of cytochrome c oxidase to maintain mitochondrial function and cellular energy supply [66]. This study showed that hepatic COX17 mRNA levels in the Cu-supplemented group decreased, which is consistent with previous theories. Cells reduce COX17 levels to prevent issues such as enhanced oxidative stress and mitochondrial dysfunction caused by excessive Cu ions [67]. The CCS transfers Cu ions to SOD1 and activates them to catalyse the disproportionation of superoxide anion radicals [68,69]. In this experiment, the changing trend of CCS mRNA levels was consistent with that of COX17. Studies have found that a long-term high-Cu diet makes rat liver Cu levels rise a lot and CCS protein expression in red blood cells decline [70,71]. Once there is a problem with the CCS in transporting Cu ions, intracellular free radicals increase significantly, resulting in damage to cell structure and function [68,69]. Later studies disclosed that excessive Cu ions can bind to CXC (C, cysteine; X, any amino acid), which disrupts CCS stability [72]. ATOX1 is crucial in liver cell Cu ion transport. It specifically binds with Cu ions to create the ATOX1–Cu complex, which links Cu ions and Cu-containing proteins and guarantees the unimpeded transport of Cu ions to the Golgi [73]. In this study, hepatic ATOX1 mRNA levels in the Cu-supplemented groups were significantly higher than those in the control group. Relevant data have shown that the absence of intracellular ATOX1 leads to abnormal accumulation of Cu ions in cells [74]. ATOX1 level upregulation is an important adaptive mechanism for Cu homeostasis and liver cell integrity in a high-Cu environment. However, when the body senses changes in the intracellular Cu ion concentration, some Cu ions bind to intracellular and extracellular Cu transporters (such as ATP7A, ATP7B, and CP). ATP7A is crucial for Cu absorption and transport to Golgi cuproenzymes. In this study, different Cu levels had no effect on the ATP7A mRNA expression in the pig liver. Relevant studies have shown that ATP7A is mainly expressed in tissues such as the intestine rather than in the liver, where it is replaced by ATP7B [75,76]. A study has shown that a large amount of Cu in hepatocytes enters CP through the Atox1/ATP7B/Golgi pathway [77]. ATP7B is highly expressed in the liver and coordinates the movement of Cu from the cytoplasm to various compartments or for export out of the liver cells when needed. When the level of cellular Cu becomes elevated, the excessive Cu is transported by ATP7B into the vesicles, which then fuse and export the Cu. Thus, ATP7B regulates vesicular storage of Cu [78]. In this study, ATP7B mRNA expression levels significantly increased with 120 mg/kg DM Cu supplementation and then decreased at 240 mg/kg DM Cu. A previous study also observed significant upregulation in the mRNA expression of ATP7B proteins with similar supplemental amounts [79]. ATP7B is key to CP synthesis, transporting Cu for binding to CP precursors and their maturation. CP, an important extracellular Cu transporter, is produced in the liver. It is secreted into the blood. Its functions include transporting Cu to other tissues or organs and oxidizing Fe (II) into Fe (III), which can facilitate the absorption and mobilization of iron [80,81]. In the present study, the expression trend of CP mRNA was similar to that of ATP7B. MT is a protein rich in cysteine with a strong affinity for Cu ions. It functions as a Cu ion reservoir and participates in regulating Cu ion homeostasis [82]. Xu et al. [83] found that moderate Cu deficiency in young rats lowered hepatic MT levels and reduced the resistance to hepatotoxins. In this study, the expression of MT mRNA in the livers of growing pigs also showed a trend of first increasing and then decreasing. This indicates that adding an appropriate amount of Cu promotes its expression. Cellularly, the increased MT expression helps liver cells deal with Cu ion concentration changes, bind more Cu ions, keep intracellular free Cu concentration proper, prevent excessive Cu ion toxicity, reduce oxidative stress, and lower liver disease risk [84,85]. In this experiment, the mRNA expression of CTR1, ATP7B, CP, ATOX1, and MT was slightly higher in the Cu-AA groups than in the CuSO_4_ groups, it indicated that the difference among Cu sources was not significant. A possible reason for this is that Cu transport proteins in the liver are universal and mainly recognise Cu ions rather than their precursor compounds. For example, CTR1 mainly transports extracellular Cu ions into cells, and its affinity for Cu ions depends on their charge and radius [86]. When Cu ions reach the vicinity of liver cells, the transport factors operate according to their mechanisms and do not show significant differences owing to different Cu sources. When Cu ion concentration increases, liver transport proteins’ activity is regulated by negative feedback regardless of Cu sources, preventing excessive accumulation, ensuring stable transport for different Cu sources, and showing no performance difference. Therefore, Cu-AA can regulate gene expression to balance Cu metabolism. Its Cu transport mechanism has similarities with other Cu sources, making it convenient to change Cu sources in pig farming. Nevertheless, the drawbacks of Cu-AA in the aquaculture industry cannot be ignored. Compared with inorganic Cu, Cu-AA is relatively new, and people’s understanding and research of it are limited. Additionally, the metabolism of organic Cu in pigs is complex, and the potential impacts of its metabolites on the organism and the environment still await further research. The limitations of this study are as follows. It mainly focuses on a specific pig breed and age stage, only involving a single growth stage. Additionally, the universality of liver Cu transport proteins conceals the differences among Cu sources, restricting the generalizability of the research results. In future research, the Cu requirements and metabolic changes of pigs in different physiological stages should be explored. A multi-omics approach should be integrated to comprehensively analyse the impacts on multiple tissues. The post-translational modifications and subcellular localizations of Cu transport proteins need to be studied. Also, the relationship between Cu and other trace elements, as well as the effects of organic addition forms, should be investigated. Meanwhile, environmental factors such as the temperature, humidity and hygiene conditions of the breeding environment and water quality, which may interfere with the Cu metabolism of pigs, should be taken into account. By integrating these aspects, a scientific basis and guidance for the application of Cu in pig farming can be provided, thereby promoting the sustainable development of the pig industry.

## 5. Conclusions

In conclusion, under the experimental conditions of this study, compared with CuSO_4_, Cu-AA can effectively increase the final weight and ADG of growing pigs. At the same time, it increases the Cu concentrations in muscles, liver, and liver subcellular organelles, reducing the Cu concentrations in kidneys and faeces, facilitating the efficient use of Cu and reducing Cu emissions in the environment. Compared with 240 mg/kg of Cu, 120 mg/kg of Cu could significantly promote the increase in the mRNA expression levels of CTR1, ATP7B, CP, ATOX1, and MT in the livers of pigs. In contrast, the mRNA expression levels of COX17 and CCS decreased. This indicates that adding an appropriate amount of Cu is more conducive to regulating the gene expression of proteins related to Cu metabolism in pig livers. The insignificant differences in the influence of different Cu sources on the gene expression of proteins related to Cu metabolism in pig livers suggest that liver Cu transporters have universality in the recognition and transport of Cu ions, and the transport mechanism may mainly depend on the Cu ions themselves rather than the specific forms of Cu sources. In our subsequent work, we prefer to use organic Cu as a Cu supplement and further explore the Cu addition levels according to the different physiological stages of animals and production objectives. This research achievement provides a theoretical support for the selection of Cu sources in the pig farming industry under the premise of considering productivity balance. Practitioners can accurately select suitable Cu sources and optimize feed formulations based on the growth requirements of pigs, costs, and expected benefits, promoting the development of the pig farming industry towards a more efficient and scientific modernization.

## Figures and Tables

**Table 1:** Composition of basal diet.

Ingredient, %	Factors and Treatments ^a^			
	AM	AH	BM	BH
Corn	60	60	60	60
Soybean meal	23	23	23	23
Corn germ meal	5	5	5	5
Wheat bran	6.35	6.35	6.35	6.35
Fish meal	2.00	2.00	2.00	2.00
Limestone	0.50	0.50	0.50	0.50
NaCl	0.35	0.35	0.35	0.35
Ca(H_2_PO_4_)_2_	1.80	1.80	1.80	1.80
1% Premix ^b^	1.00	1.00	1.00	1.00
Total	100.00	100.00	100.00	100.00
Nutritional composition				
Digestible energy (MJ/kg) ^c^	13.65	13.65	13.65	13.65
Crude protein	17.45	17.45	17.45	17.45
Ca (%) ^d^	0.76	0.76	0.76	0.76
P (%) ^d^	0.47	0.47	0.47	0.47
Lysine (%) ^d^	1.19	1.19	1.19	1.19
Methionine (%) ^d^	0.48	0.48	0.48	0.48
Cu (mg/kg) ^d^	119.23	241.67	120.15	240.06

^a^ AM: diet supplemented with CuSO_4_ (120 mg/kg DM); AH: CuSO_4_ (240 mg/kg DM); BM: Cu-AA (120 mg/kg DM) and BH: Cu-AA (240 mg/kg DM) diet. ^b^ Per kilogram of diet provided: 150 mg of Fe, 200 mg of Zn, 10 mg of Mn, 0.15 mg of I (potassium iodide), 0.30 mg of Se (NaSeO_3_·H_2_O), 4400 IU of vitamin A, 440 IU of vitamin D, 22 IU of vitamin E, 1.1 mg of vitamin K (menadione dimethylpyridine bisulphate), 4.4 mg of riboflavin, 22 mg of D-pantothenic acid, 22 mg of niacin, 22 µg of vitamin B_12_, 0.61 g of choline (choline chloride), 0.14 mg of D-biotin, and 0.66 mg of folic acid. ^c^ Calculated according to NRC (2012). ^d^ Measured value.

**Table 2:** Effects of Cu sources and levels on the growth performance of growing pigs.

Items	Cu Source (CS)		Cu Level (CL) mg/kg		Treatment				SEM	*p* Value		
	CuSO_4_ (A)	Cu-AA (B)	120 mg/kg (M)	240 mg/kg (H)	AM	AH	BM	BH		CS	CL	CS × CL
initial weight (kg)	14.130	14.200	14.260	14.070	14.300	13.960	14.220	14.180	0.039	0.557	0.123	0.217
Final weight (kg)	37.141	37.599	37.449	37.291	37.358 ^ab^	36.924 ^b^	37.540 ^a^	37.658 ^a^	0.087	0.008	0.312	0.087
ADFI (kg/d)	1.162	1.173	1.164	1.171	1.164	1.160	1.164	1.182	0.003	0.126	0.320	0.126
ADG (kg/d)	0.575	0.584	0.579	0.581	0.576 ^b^	0.574 ^b^	0.583 ^ab^	0.587 ^a^	0.002	0.009	0.809	0.348
F: G	2.022	2.004	2.008	2.018	2.022	2.021	1.994	2.014	0.004	0.168	0.434	0.434

Note: Mean difference in individual effect of copper source, copper level, and different treatments in the same line (*p* < 0.05). For inter-factor group comparisons, distinct superscripts denote significant differences (*p* < 0.05). Abbreviations: ADFI, average daily feed intake; ADG, average daily gain; F: G, feed conversion ratio.

**Table 3:** Effects of Cu sources and levels on the serum Cu concentrations of growing pigs.

Items	Cu Source (CS)		Cu Level (CL) mg/kg		Treatment				SEM	*p* Value		
	CuSO_4_ (A)	Cu-AA (B)	120 mg/kg (M)	240 mg/kg (H)	AM	AH	BM	BH		CS	CL	CS × CL
10d (mg/L)	1.332	1.336	1.332	1.336	1.320	1.344	1.344	1.328	0.003	0.921	0.921	0.621
20d (mg/L)	1.612	1.596	1.580	1.628	1.576 ^b^	1.648 ^a^	1.584 ^b^	1.608 ^ab^	0.009	0.405	0.021	0.218
40d (mg/L)	2.030	2.071	1.711	2.390	1.694 ^b^	2.366 ^a^	1.728 ^b^	2.414 ^a^	0.111	0.082	<0.001	0.755

Note: Mean difference in individual effect of copper source, copper level, and different treatments in the same line (*p* < 0.05). For inter-factor group comparisons, distinct superscripts denote significant differences (*p* < 0.05).

**Table 4:** Effects of Cu sources and levels on the tissue Cu concentrations of growing pigs.

Items	Cu Source (CS)		Cu Level (CL) mg/kg		Treatment				SEM	*p* Value		
	CuSO_4_ (A)	Cu-AA (B)	120 mg/kg (M)	240 mg/kg (H)	AM	AH	BM	BH		CS	CL	CS × CL
Liver (mg/kg)	20.484	21.932	15.114	27.302	14.518 ^d^	26.450 ^b^	15.710 ^c^	28.154 ^a^	2.009	<0.001	<0.001	0.077
Kidney (mg/kg)	17.191	16.087	13.389	19.889	14.500 ^b^	19.882 ^a^	12.278 ^c^	19.896 ^a^	1.089	<0.001	<0.001	<0.001
Muscle (mg/kg)	3.766	5.218	3.994	4.990	4.220 ^b^	3.312 ^d^	3.768 ^c^	6.668 ^a^	0.384	<0.001	<0.001	<0.001
Faeces (mg/kg)	1566.120	1557.284	1212.015	1911.389	1212.330 ^c^	1919.910 ^a^	1211.670 ^c^	1911.389 ^b^	114.935	<0.001	<0.001	<0.001

Note: Mean difference in individual effect of copper source, copper level, and different treatments in the same line (*p* < 0.05). For inter-factor group comparisons, distinct superscripts denote significant differences (*p* < 0.05).

**Table 5:** Effects of Cu sources and levels on the blood biochemical parameters of growing pigs.

Items		Cu Source (CS)		Cu Level (CL) mg/kg		Treatment				SEM	*p* Value		
		CuSO_4_ (A)	Cu-AA (B)	120 mg/kg (M)	240 mg/kg (H)	AM	AH	BM	BH		CS	CL	CS × CL
ALB (g/L)	10d	36.311	37.553	36.511	37.353	35.378	37.243	37.643	37.462	0.281	0.102	0.257	0.172
	20d	43.380	48.371	41.987	49.763	38.735 ^d^	48.025 ^b^	45.239 ^c^	51.502 ^a^	1.526	<0.001	<0.001	0.023
	40d	50.030	52.961	52.662	50.330	50.719 ^b^	49.341 ^b^	54.605 ^a^	51.318 ^b^	0.626	<0.001	<0.001	0.042
TP (g/L)	10d	61.061	63.602	61.322	63.341	58.523 ^b^	63.600 ^a^	64.121 ^a^	63.083 ^a^	0.670	0.060	0.127	0.027
	20d	64.873	66.148	65.990	65.032	65.063	64.683	66.916	65.380	0.272	0.099	0.206	0.438
	40d	78.383	81.130	77.004	82.510	74.845 ^c^	81.921 ^a^	79.162 ^b^	83.098 ^a^	1.029	0.003	<0.001	0.067
AST (U/L)	10d	41.114	42.264	41.364	42.314	40.884	41.345	41.845	42.684	0.218	0.107	0.349	0.765
	20d	46.209	46.487	43.975	48.721	43.955 ^b^	48.463 ^a^	43.996 ^b^	48.978 ^a^	0.779	0.800	<0.001	0.829
	40d	52.513	53.501	46.252	59.763	45.002 ^c^	60.024 ^a^	47.501 ^b^	59.501 ^a^	2.226	0.035	<0.001	0.003
ALT (U/L)	10d	47.114	46.045	47.987	45.171	49.412 ^a^	44.816 ^c^	46.564 ^b^	45.526 ^c^	0.521	0.079	<0.001	0.007
	20d	57.314	56.501	58.412	55.402	59.424 ^a^	55.204 ^c^	57.400 ^b^	55.601 ^c^	0.535	0.070	<0.001	0.011
	40d	77.803	83.703	79.104	82.403	75.404 ^b^	80.202 ^ab^	82.803 ^a^	84.603 ^a^	1.124	0.003	0.073	0.396

Note: Mean difference in individual effect of copper source, copper level, and different treatments in the same line (*p* < 0.05). For inter-factor group comparisons, distinct superscripts denote significant differences (*p* < 0.05). Abbreviations: ALB, albumin; TP, total protein; AST, aspartate aminotransferase; ALT, alanine aminotransferase.

**Table 6:** Effects of Cu sources and levels on liver subcellular Cu concentrations.

Items	Cu Source (CS)		Cu Level (CL) mg/kg		Treatment				SEM	*p* Value		
	CuSO_4_ (A)	Cu-AA (B)	120 mg/kg (M)	240 mg/kg (H)	AM	AH	BM	BH		CS	CL	CS × CL
Mitochondria (%)	6.658	6.928	6.357	7.229	6.180 ^c^	7.136 ^a^	6.534 ^b^	7.322 ^a^	0.150	0.006	<0.001	0.340
Lysosome (%)	9.951	10.252	9.730	10.473	9.682 ^c^	10.220 ^b^	9.778 ^c^	10.726 ^a^	0.134	0.042	<0.001	0.151
Cytoplasm (%)	46.952	47.636	44.394	50.494	43.480 ^c^	50.424 ^a^	45.308 ^b^	50.564 ^a^	1.016	0.021	<0.001	0.043

Note: Mean difference in individual effect of copper source, copper level, and different treatments in the same line (*p* < 0.05). For inter-factor group comparisons, distinct superscripts denote significant differences (*p* < 0.05).

**Table 7:** Effects of Cu sources and levels on the activities of related enzymes in liver subcellular organelles.

Items		Cu Source (CS)		Cu Level (CL) mg/kg		Treatment				SEM	*p* Value		
		CuSO_4_ (A)	Cu-AA (B)	120 mg/kg (M)	240 mg/kg (H)	AM	AH	BM	BH		CS	CL	CS × CL
CTSD (pg/mL)	Lysosome	86.354	86.257	81.292	91.319	81.463 ^b^	91.246 ^a^	81.123 ^b^	91.39 1 ^a^	1.641	0.906	<0.001	0.769
	Cytoplasm	87.301	87.730	85.553	91.478	83.393 ^b^	91.210 ^a^	83.714 ^b^	91.746 ^a^	1.211	0.417	<0.001	0.838
BGL (ng/mL)	Lysosome	33.373	33.351	31.617	35.106	31.540 ^b^	35.205 ^a^	31.695 ^b^	35.007 ^a^	1.641	0.909	<0.001	0.345
	Cytoplasm	30.645	30.438	29.508	31.576	29.750 ^b^	31.540 ^a^	29.265 ^b^	31.611 ^a^	1.149	0.283	<0.001	0.152
ACP (ng/gprot)	Lysosome	54.339	54.786	55.300	53.825	54.787	53.891	55.813	53.759	0.264	0.326	0.326	0.326
	Cytoplasm	45.424	46.120	31.649	59.895	31.427 ^d^	59.421 ^b^	31.871 ^c^	60.369 ^a^	4.624	<0.001	<0.001	0.096

Note: Mean difference in individual effect of copper source, copper level, and different treatments in the same line (*p* < 0.05). For inter-factor group comparisons, distinct superscripts denote significant differences (*p* < 0.05). Abbreviations: CTSD, cathepsin D; BGL, β-glucosidase, ACP, acid phosphatase.

**Table 8:** Effects of Cu sources and levels on the mRNA expression levels of transporters in the liver.

Items	Cu Source (CS)		Cu Level (CL) mg/kg		Treatment				SEM	*p* Value		
	CuSO_4_ (A)	Cu-AA (B)	120 mg/kg (M)	240 mg/kg (H)	AM	AH	BM	BH		CS	CL	CS × CL
CTR1	1.024	1.111	1.376	0.760	1.301 ^a^	0.748 ^b^	1.452 ^a^	0.771 ^b^	1.286	0.158	<0.001	0.296
ATP7A	0.895	0.888	0.886	0.896	0.880	0.910	0.893	0.882	0.003	0.711	0.636	0.343
ATP7B	1.325	1.379	1.886	0.818	1.825 ^a^	0.825 ^b^	1.945 ^a^	0.812 ^b^	0.175	0.495	<0.001	0.396
CP	2.027	2.061	2.381	1.707	2.331 ^a^	1.722 ^b^	2.431 ^a^	1.692 ^b^	0.110	0.634	<0.001	0.378
MT	2.423	2.437	2.756	2.104	2.731 ^a^	2.115 ^b^	2.780 ^a^	2.094 ^b^	0.106	0.818	<0.001	0.565
ATOX1	1.043	1.043	1.053	1.032	1.065	1.021	1.042	1.0434	0.004	0.985	0.349	0.314
CCS	0.906	0.894	0.902	0.897	0.913	0.898	0.892	0.896	0.002	0.323	0.670	0.438
COX17	0.941	0.959	0.947	0.953	0.941	0.941	0.953	0.965	0.003	0.271	0.702	0.704

Note: Mean difference in individual effect of copper source, copper level, and different treatments in the same line (*p* < 0.05). For inter-factor group comparisons, distinct superscripts denote significant differences (*p* < 0.05). Abbreviations: CTR1, copper transporter protein 1; ATP7B, ATPase copper-transporting beta; CP, ceruloplasmin; ATOX1, antioxidant protein 1; MT, metallothionein; ATP7A, ATPase copper-transporting alpha; COX17, cytochrome c oxidase copper chaperone 17; CCS, copper chaperone for superoxide dismutase.

## Data Availability

The original contributions of this study are in this article. For further inquiries, contact the corresponding authors.
